# Development and application of a dual LAMP-LFD assay for the simultaneous detection of *Streptococcus suis* and *Glaesserella parasuis*

**DOI:** 10.3389/fcimb.2025.1575365

**Published:** 2025-04-01

**Authors:** Haojie Wang, Chenhui Dong, Xiaoxiao Tian, Yao Pan, Longxi Wang, Tonqging An, Liangquan Zhu

**Affiliations:** ^1^ State Key Laboratory for Animal Disease Control and Prevention, Harbin Veterinary Research Institute, Chinese Academy of Agricultural Sciences, Harbin, China; ^2^ China Institute of Veterinary Drug Control, Beijing, China; ^3^ Animal Husbandry Science Institute of Ganzi Tibetan Autonomous Prefecture, Kangding, China

**Keywords:** *Streptococcus suis*, *Glaesserella parasuis*, LAMP, LFD, rapid detection

## Abstract

**Introduction:**

*Streptococcus suis* (*S. suis*) and *Glaesserella parasuis* (*G. parasuis*) are prevalent pathogens in pig populations and are often associated with co-infections, leading to substantial economic losses in the swine industry. However, there is currently a shortage of rapid detection methods. In this study, a dual loop-mediated isothermal amplification combined with lateral flow dipstick (LAMP-LFD) assay was developed for the simultaneous and convenient detection of S. suis and G. parasuis.

**Methods:**

The assay utilized primers targeting the conserved regions of the gdh gene of S. suis and the infB gene of *G. parasuis*. Optimal primer sets were identified, and reaction conditions, including temperature, time, and primer concentration ratios, were optimized using single-variable control method. The LAMP-LFD assay was established with biotin and digoxin or biotin and 6-FAM-labeled FIP/BIP primers, combined with LFD.

**Results:**

The assay was most effective at a reaction temperature of 62°C, a primer concentration ratio of 1:4, and a reaction time of 40 minutes. The minimum detection limits were 22 and 18 copies/μL for recombinant plasmids and 19 and 20 CFU for bacterial samples of S. suis and G. parasuis, respectively. The assay showed no cross-reactivity with other pathogens and exhibited high adaptability across various thermal platforms, including PCR instruments, metal baths, and water baths. Clinical testing of 106 samples revealed positive rates of 11.32% (12/106) for S. suis, 25.47% (27/106) for *G. parasuis*, and 2.83% (3/106) for mixed infections.

**Discussion:**

This simple, rapid, specific, and sensitive dual LAMP-LFD assay provides robust technical support for the prevention and control of swine streptococcosis and Glässer's disease.

## 1 Introduction

*Streptococcus suis* (*S. suis*, SS) and *Glaesserella parasuis* (*G. parasuis*, GPS) are significant and prevalent pathogens in swine, causing similar clinical symptoms and pathological lesion (Guo et al., 2024; Santoya et al., 2024). Both pathogens can lead to septicemia, polyserositis, meningitis, arthritis, and pneumonia (Jiang et al., 2024; Yue et al., 2024). *S. suis* is classified into 35 serotypes based on capsular polysaccharides, with serotypes 1, 2, 1/2, 7, 9, and 14 being particularly pathogenic in swine, and serotype 2 being the most widespread and severe (Fan et al., 2024; Petrocchi et al., 2024; Xia et al., 2024). In contrast, *G. parasuis* has 15 serotypes, with serotypes 4, 5, and 12 most frequently isolated from clinical cases, though serotypes 1, 2, 6, 7, 9, and 14 have also been reported (Schuwerk et al., 2020; Wu et al., 2023; Yan et al., 2023). Cross-protection between serotypes is limited (Bujold et al., 2023). Co-infections of *S. suis* and *G. parasuis* are common and pose a significant clinical challenge due to overlapping symptoms. In recent years, *S. suis* and *G. parasuis* infections have been widely prevalent in the global swine industry, emerging as major pathogens that severely impact pig health. In high-density pig farming regions of Italy, *S. suis* and *G. parasuis* were responsible for 18.0% and 20.3% of bacterial arthritis cases in weaned piglets, respectively (Salogni et al., 2022). Epidemiological studies conducted in Austria (2016–2021) and the United States (2017–2022) reported *S. suis* and *G. parasuis* co-infection rates of 3% and 5%, respectively (Renzhammer et al., 2023; Silva et al., 2023). In contrast, the prevalence in China appears to be higher. A study in the eastern region of China revealed *S. suis* and *G. parasuis* detection rates of 52.3% and 33.2%, with a co-infection rate of 33.2% (Zhu et al., 2021). Additionally, a study in Heilongjiang province using multiplex real-time quantitative PCR found that *S. suis* serotype 2 and *G. parasuis* detection rates exceeded 60%, with co-infection rates ranging from 78% to 96%. However, the limited sample size may not accurately reflect the broader epidemiological situation (Li et al., 2024). These findings indicate that *S. suis* and *G. parasuis* co-infections are common in the swine industry, highlighting the critical need to develop rapid, sensitive, and specific diagnostic methods for effective disease prevention and control.

Current diagnostic methods for *S. suis* and *G. parasuis* include bacterial isolation, molecular biology techniques, and serological assays (Nedbalcova et al., 2022; de Jong et al., 2023). However, bacterial isolation is labor-intensive, time-consuming, and less practical in clinical settings (Scherrer et al., 2024). Molecular techniques such as conventional PCR and quantitative PCR provide high specificity and sensitivity but require expensive equipment and skilled personnel, limiting their application in field conditions (Goto et al., 2023; Wang et al., 2024). Serological assays, including agglutination tests and ELISA, are hindered by high material costs and their inability to simultaneously detect both pathogens (Guo et al., 2010; Chidkoksung et al., 2024).

Compared to conventional PCR and qPCR methods, LAMP technology does not require expensive thermal cyclers, offering lower costs and shorter detection times, while maintaining high sensitivity and strong specificity. Particularly, when combined with LFD, LAMP enables rapid on-site detection, facilitating timely intervention and disease control. This makes the LAMP-LFD approach highly promising for applications in disease diagnostics (Pilchova et al., 2020; Bai et al., 2024). To date, no dual LAMP-LFD detection method has been developed for *S. suis* and *G. parasuis*. This study aims to establish a dual LAMP-LFD assay for the simultaneous detection of these pathogens in pigs, improving diagnostic accuracy, facilitating epidemiological investigations, and enhancing the prevention and control of swine streptococcosis and Glässer’s disease.

## 2 Materials and methods

### 2.1 Bacterial strains and clinical samples

*S. suis* serotype 1 CVCC2937 strain, serotype 2 CVCC9740 strain, serotype 7 CVCC563 strain, serotype 9 CVCC989 strain, serotype 14 CVCC212 strain, serotype 16 CVCC223 strain, and *Mycoplasma hyopneumoniae* CVCC679, and *G. parasuis* serotype 4 CVCC156 strain, serotype 5 CVCC167 strain, serotype 12 CVCC134 strain, and *Enterococcus faecalis* CVCC1927 strain, *Streptococcus agalactiae* CVCC586 strain, *Pasteurella multocida* CVCC390 strain, *Actinobacillus pleuropneumoniae* CVCC259 strain, *Streptococcus pyogenes* CVCC593 strain, and *Streptococcus pneumoniae* CVCC1929 strain were stored and supplied by China Institute of Veterinary Drug Control. From July 2023 to December 2024, 106 clinical samples (pleural effusion, lung tissue, nasal swabs) with respiratory disease and arthritis symptoms were collected from pig farms in Henan province.

### 2.2 Primer design and screening

Primers targeting the conserved regions of the *S. suis gdh* gene (AM946016.1) and the *G. parasuis infB* gene (CP071489.1) were designed following the principles of LAMP primer design by using Primer Explorer V5 software (http://primerexplorer.jp/lampv5e/index.html) (Kirkoyun et al., 2024). The fluorescent dye method was employed to identify the most specific and effective primer sets.

The LAMP reaction was performed in a 25 μL system comprising 5 μL of 5 × LAMP Reaction Mix, 2 μL of *Bst* II DNA polymerase (Harbin Tianyuehao Biotechnology Co., Ltd), 0.45 μL of TS LAMP Green (20×), 1.6 μM of FIP/BIP, 0.4 μM of B3/F3, 2 μL of DNA template, and ddH_2_O to a final volume of 25 μL. The reaction was carried out at 62°C for 40 cycles, with fluorescence signals recorded every minute. Two optimal primer pairs were selected and labeled at the 5’ ends with Biotin and 6-FAM, and Biotin and Digoxigenin, respectively (**Table 1**). Additionally, primers for standard plasmids were designed using Primer Premier 5 software (**Table 1**). All primers, including the labeled ones, were synthesized by Beijing Liuhehuada Gene Technology Co., Ltd.

**Table 1:** Primers for *S. suis* and *G. parasuis* used in this study.

Pathogen	Primers	Sequence (5’-3’)
*S. suis*	SS-F3	GGCAATCATGCTATCCGCAA
	SS-B3	CTCGCAAAAGCTGCCAAC
	SS-FIP	Biotin**-**GGCCAACATCTTCAACACAGCCGTTAGCACCTGCAAGGTAGT
	SS-BIP	6-FAM**-**ACGAGTCCATGACAAGCGAAGGGGTGGTGTAGCTGTATCTGC
	gdh-F	TTATACCAAACCTTGGGCAATCA
	gdh-R	ATGTCAAATGCCAAAGCTTACATC
*G. parasuis*	GPS-F3	ACCACCGAATTTCTCAGAA
	GPS-B3	CGAAAGCAACGGATATCGT
	GPS-FIP	Biotin**-**TGCGGTAAACAAAATTGATAAACCAATCACTTCGTGTTGTAATAACTCT
	GPS-BIP	Digoxigenin**-**TTCGCGTGTTGGATTGCTTCTTGTAGTAGCAGCTGACG
	infB-F	GTTACGGACTTCTGAAACGTCGT
	infB-R	CTCATTATTAGACTATATCCGTAAAGCGA

### 2.3 Construction of recombinant plasmid standards

Genomic DNA from *S. suis* and *G. parasuis* was used as a template to design two pairs of specific primers for single PCR amplification. The PCR products were analyzed by 1% agarose gel electrophoresis, purified using the EasyPure^®^ Quick Gel Extraction Kit (TransGen Biotech), and subsequently cloned into the pMD18-T vector to construct recombinant plasmids, designated as pMD-SS and pMD-GPS. The recombinant plasmids were verified by PCR and sequencing. Plasmid extraction was performed using a plasmid extraction kit, and the plasmid concentrations were accurately determined. The number of plasmid copies was calculated using the following equation: (Plasmid copies/µL = (6.02×10^23^) × [X^*^ ng/µL×10^−9^)/constructed plasmid length (bp) × 660] (Wang et al., 2023).

X* means recombinant plasmid concentration.

### 2.4 Optimization of the LAMP reaction conditions

The single-variable control method was employed to optimize reaction conditions, including temperature, time, and primer concentration ratio. Reaction temperatures were tested at 60.0, 60.3, 61.0, 62.0, 63.2, 64.2, 64.7, and 65.0°C. Reaction times were evaluated at 20, 25, 30, 35, 40, and 45 minutes. Primer concentration ratios for internal to external primers (F3:FIP and B3:BIP) were assessed at 1:1, 1:2, 1:4, 1:6, 1:8, and 1:10. After each reaction, the products were analyzed by 1.5% agarose gel electrophoresis to identify and confirm the optimal conditions.

### 2.5 Specificity test

The nucleic acids of *S. suis* serotype 1, 2, 7, 9, 14, 16, *Mycoplasma hyopneumoniae*, *G. parasuis* serotype 4, serotype 5, serotype 12, *Enterococcus faecalis*, *Streptococcus agalactiae*, *Pasteurella multocida*, *Actinobacillus pleuropneumoniae*, *Streptococcus pyogenes*, and *Streptococcus pneumoniae* were used as templates for dual LAMP-LFD detection. Taking the mixture of recombinant plasmids pMD-SS and pMD-GPS as positive control, the specificity of the dual LAMP-LFD method was verified.

### 2.6 Sensitivity test

#### 2.6.1 Minimum detection limit for recombinant plasmid standards

The two positive plasmids (pMD-SS: 2.2×10^10^ copies/μL; pMD-GPS: 1.8×10^10^ copies/μL) from 10^10^ copies/μL to 10^0^ copy/μL, and the minimum copy number of the plasmids was determined by selecting 10^6^-10^0^ copies/μL for the double LAMP-LFD assay.

#### 2.6.2 Minimum number of bacteria detection

*S. suis* was cultured in 10 mL of tryptic soy broth (TSB) supplemented with 5% neonatal bovine serum, while *G. parasuis* was cultured in 10 mL of TSB supplemented with 5% neonatal bovine serum and 0.1% NAD. Both bacterial cultures were incubated at 37°C with shaking at 180 rpm for 12 hours and then serially diluted up to 10^-7^. From each dilution (10^-5^, 10^-6^, 10^-7^), 100 μL of the bacterial suspension was plated onto tryptic soy agar (TSA) containing 5% neonatal bovine serum and 0.1% NAD. The bacterial suspensions were evenly spread using a sterile spreader, and colony counts were recorded after 12 hours of incubation at 37°C. This procedure was performed in triplicate. Additionally, 0.25 g of healthy porcine lung tissue was homogenized, and 100 μL of the diluted bacterial suspension was added to the homogenate. Genomic DNA was extracted using a commercial DNA extraction kit and subsequently analyzed using the dual LAMP-LFD assay.

### 2.7 Repeatability and suitability tests

Three batches of diagnostic reagents were prepared to evaluate inter- and intra-batch sensitivity, specificity, and reproducibility. Thermostatic reactions were performed using these reagents across three platforms: a PCR instrument, a metal bath, and a water bath, to assess the compatibility and suitability of each instrument for the assays.

### 2.8 Clinical application

106 clinical samples, including pleural effusion, lung tissue, nasal swabs, and others collected from pigs, were tested using the LAMP-LFD method. These samples were also tested using an association standard (GB/T 19915.5—2005, Protocol of multiplex PCR identification of *Streptococcus suis* type 2; GB/T 34750—2017, Detection methods for *haemophilus parasuis*) to validate the feasibility of the LAMP-LFD method (Xin et al., 2023).

## 3 Results

### 3.1 Construction of recombinant plasmids

*S. suis* and *G. parasuis* genomic DNA were used as templates for PCR amplification with two pairs of specific primers designed for each pathogen, respectively. The PCR products were then ligated into the pMD18-T vector to generate the recombinant plasmids pMD-SS or pMD-GPS. These plasmids were verified by PCR amplification and DNA sequencing, confirming that the amplified target fragments matched the expected sequences (**Supplementary Figure S1**). The concentrations of the recombinant plasmids were determined to be 97.42 ng/μL and 79.96 ng/μL, corresponding to 2.2 × 10^10^ copies/μL and 1.8 × 10^10^ copies/μL, respectively. The plasmids were aliquoted and stored at -20°C for future use.

### 3.2 LAMP primer screening

**Figure 1:**
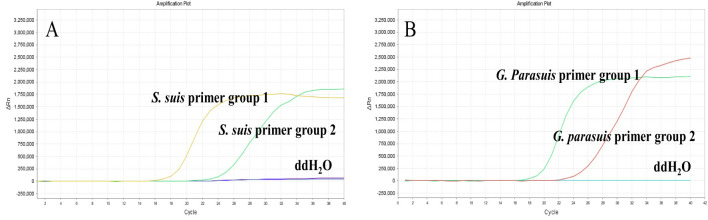
*S. suis* and *G*. *parasuis* LAMP primer screening. **(A)** the amplification curves of *S. suis* based on primers set 1 and set 2; **(B)** the amplification curves of *G. parasuis* based on primers set 1 and set 2.

Each of the two sets of LAMP primers designed in this study was tested separately using a fluorescence quantitative PCR instrument. The results demonstrated that all four primer sets produced positive amplification curves, while negative controls showed no amplification (**Figure 1**). Under the same conditions, primer set 1 for *S. suis* (**Figure 1A**) and primer set 1 for *G. parasuis* (**Figure 1B**) exhibited smoother curves with lower Ct values and higher amplification efficiency. Consequently, *S. suis* primer set 1 and *G. parasuis* primer set 1 were selected for use in the dual LAMP-LFD assay (**Table 1**).

### 3.3 Conditional optimization, final reaction conditions, and result determination

**Figure 2:**
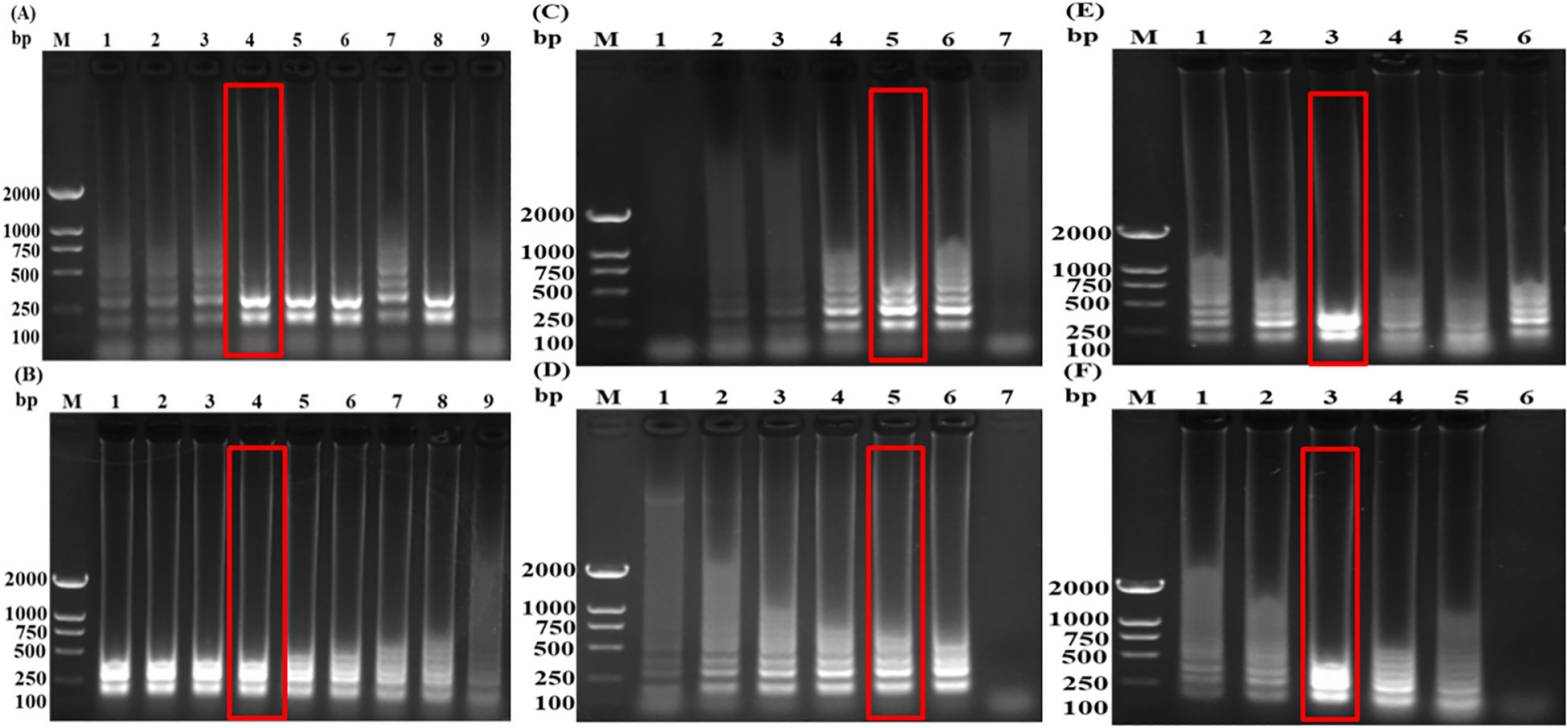
Optimization of *S. suis* **(A, C, E)** and *G*. *parasuis* **(B, D, F)** LAMP reaction conditions. M: DL 2000 DNA Marker; **(A)** and **(B)** 1-8: 60, 60.3, 61, 62, 63.2, 64.2, 64.7, 65°C; 9: ddH_2_O; C and D: 1-6: 20, 25, 30, 35, 40, 45min; 7: ddH_2_O; **(C)** and **(D)** 1-6: 1:1, 1:2, 1:4, 1:6, 1:8, 1:10.

**Figure 3:**
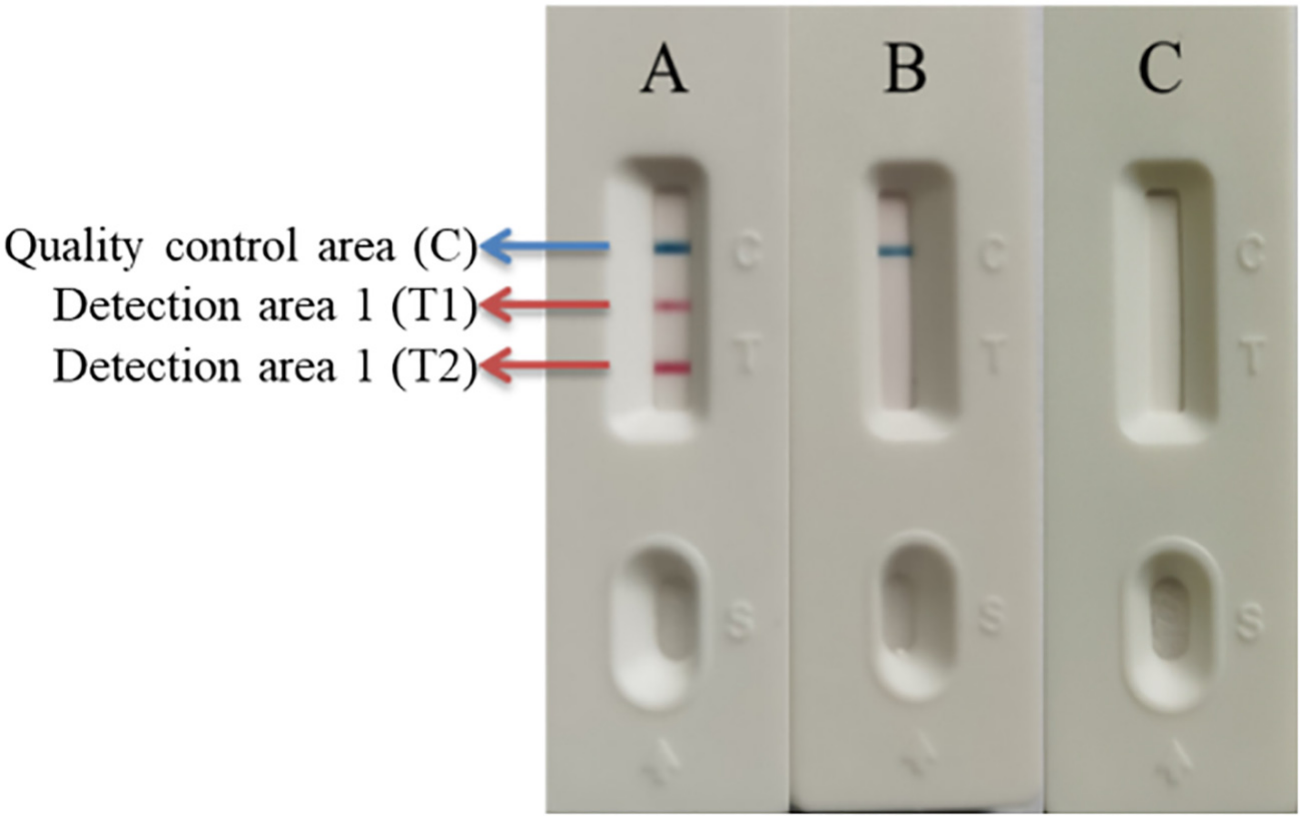
Example of double LAMP-LFD result determination. **(A)** Positive; **(B)** Negative; **(C)** Invalid.

The reaction temperature, reaction time, and primer concentration ratio were optimized using the single control variable method. The results indicated that at a reaction temperature of 62°C, the LAMP amplification products consistently exhibited clear, typical trapezoidal bands (**Figures 2A, B**). When the reaction time was 40 minutes or longer, the LAMP amplification products became stable, with clear bands observed (**Figures 2C, D**). Additionally, a primer concentration ratio of 1:4 yielded the most effective and clearly defined LAMP amplification bands (**Figures 2E, F**). The LAMP reaction system (25 μL) includes: 5× LAMP Reaction Mix 5 μL, Bst *II* DNA Polymerase 2 μL, SS-F3 (10 μM) 1 μL, SS-B3 (10 μM) 1 μL, SS-FIP (100 μM) 0.4 μL, SS-BIP (10 μM) 0.4 μL, GPS-F3 (10 μM) 1 μL, GPS-B3 (10 μM) 1 μL, GPS-FIP (100 μM) 0.4 μL, GPS-BIP (10 μM) 0.4 μL, Template DNA 2 μL, ddH_2_O 10.4 μL. The reaction was carried out at a constant temperature of 62°C for 40 minutes. After the reaction, 5-10 μL of the LAMP amplification product was diluted 20 times with ddH_2_O, mixed well, and 80 μL of the diluted reaction product was dropped onto the sample hole. The results in the detection zone were recorded within 15 minutes. The color changes in the T line and C line of the test strip were observed (**Figure 3**; **Table 2**).

**Table 2:** Determination of test strip results.

	Quality control area (C)	Detection area 1 (T1)	Detection area 2 (T2)
*S. suis*	Blue	Red	None
*G. parasuis*	Blue	None	Red
*S. suis* + *G. parasuis*	Blue	Red	Red
Negative	Blue	None	None
Invalid	None	Red	Red
Invalid	None	Red	None
Invalid	None	None	Red

### 3.4 Specificity of the dual LAMP-LFD assay

**Figure 4:**
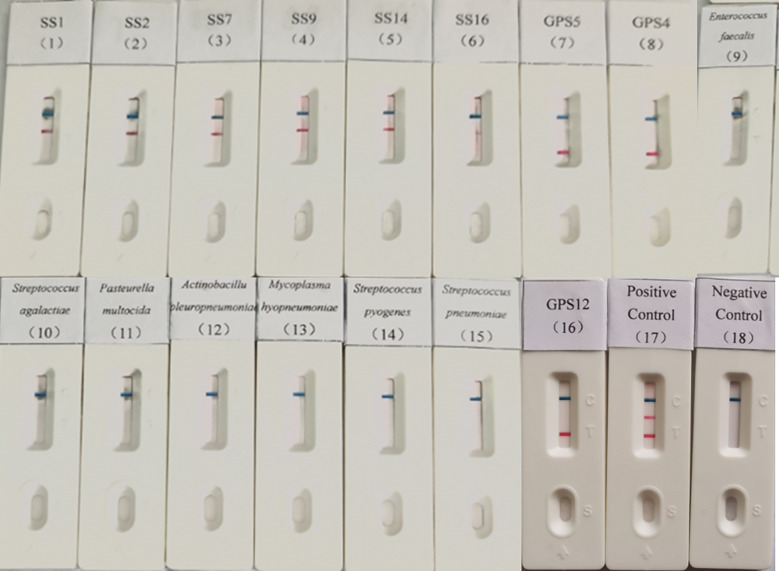
Specificity validation of dual LAMP-LFDs. 1: SS1; 2: SS2; 3: SS7; 4: SS9; 5: SS14; 6: SS16; 7: GPS5; 8: GPS4; 9: *Enterococcus faecalis*; 10: *Streptococcus agalactiae*; 11: *Pasteurella multocida*; 12: *Actinobacillus pleuropneumoniae*; 13: *Mycoplasma hyopneumoniae*; 14: *Streptococcus pyogenes*; 15: *Streptococcus pneumoniae*; 16: GPS12; 17: Positive control; 18: Negative control.

Genomic DNA of *S. suis* seotypes 1, 2, 7, 9, 14, and 16; *Mycoplasma hyopneumoniae*; *G. parasuis* types 4, 5, and 12; *Enterococcus faecalis*; *Streptococcus agalactiae*; *Pasteurella multocida*; *Actinobacillus pleuropneumoniae*; *Streptococcus pyogenes* and *Streptococcus pneumoniae* were tested using the established dual LAMP-LFD method. The results indicated only the *S. suis* and *G. parasuis* were detectable, with no cross-reactivity observed with any of the other porcine pathogens (**Figure 4**), suggesting the method is high specificity.

### 3.5 Sensitivity of the dual LAMP-LFD assay

**Figure 5:**
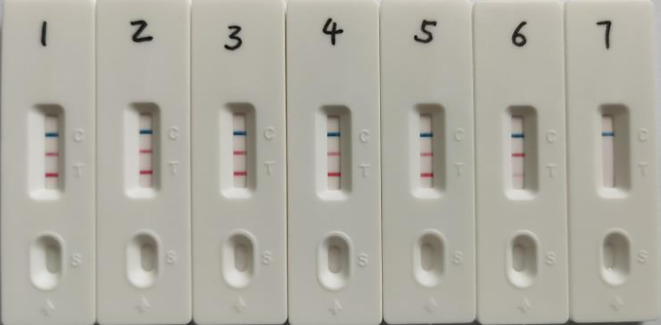
Recombinant plasmid standards as templates for dual LAMP-LFD sensitivity assay. 1-7: pMD-SS concentrations from 2.2×10^6^-2.2×10^0^ copies/μL, pMD-GPS concentrations from 1.8×10^6^-1.8×10^0^ copies/μL.

**Figure 6:**
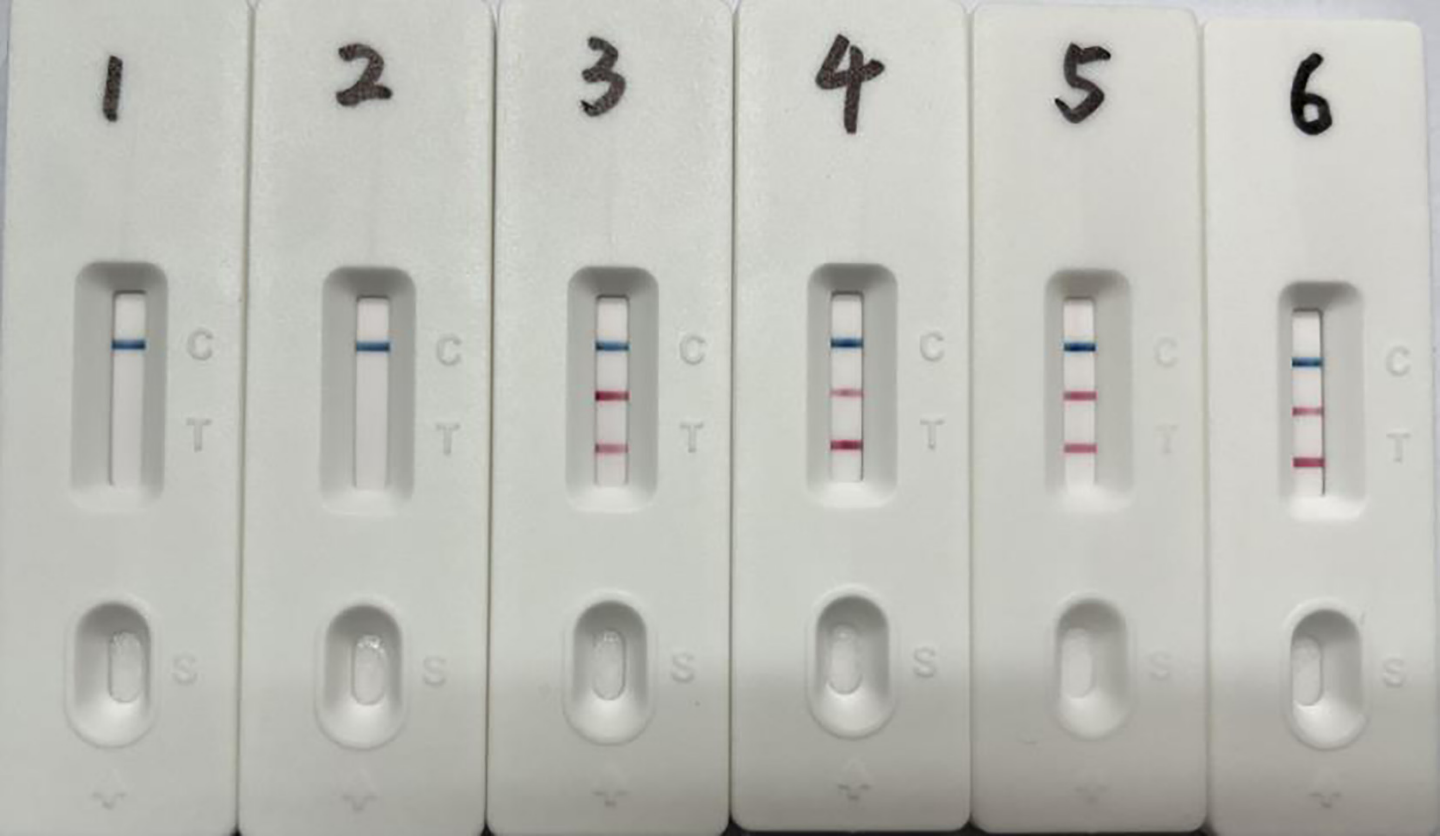
Dual LAMP-LFD detection of simulated tissue samples. 1: Pig lung tissue free of *S. suis* and *G. parasuis*; 2-6: *S. suis* 19 to 1.9 × 10^5^ CFU and *G. parasuis* 20 to 2.0 × 10^5^ CFU.

The dual LAMP-LFD assay was performed using plasmid concentrations ranging from 10^6^ copies/μL to 10^0^ copy/μL as templates to assess the minimum detectable amount of plasmid standards. The results showed that the lowest detectable copy numbers of *S. suis* and *G. parasuis* by the double LAMP-LFD method were 22 and 18 copies/μL, respectively (**Figure 5**). Bacterial counts from cultured samples were determined, revealing concentrations of 1.9 × 10⁸ CFU/mL for *S. suis* and 6 × 10⁸ CFU/mL for *G. parasuis* (**Supplementary Table S1**). The results of the dual LAMP-LFD assay demonstrated that the lowest detectable numbers of bacteria were 19 CFU/mL for *S. suis* and 20 CFU/mL for *G. parasuis* (**Figure 6**).

### 3.6 Repeatability and stability test

**Figure 7:**
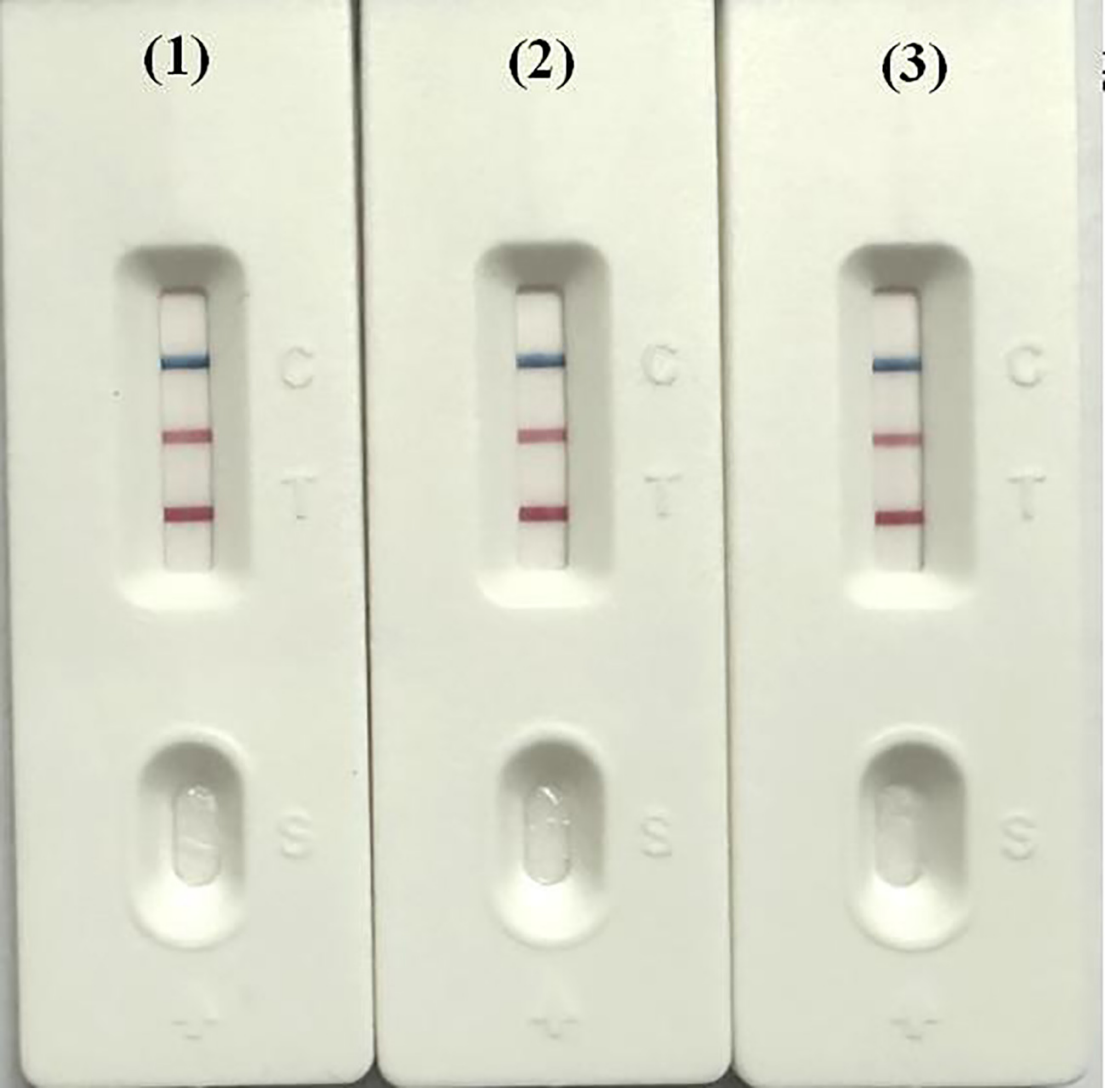
Detection of dual LAMP-LFD in different instruments. 1: PCR instrument (62°C); 2: metal bath (62°C); 3: water bath (62°C).

Three batches of diagnostic reagents were prepared and tested to assess both inter-batch and intra-batch reproducibility. Reactions using the same batch of reagents were performed on a PCR instrument, metal bath, and water bath at a temperature of 62°C to evaluate the performance across these three platforms. The results showed consistent performance in both inter- and intra-batch tests, confirming the method’s reproducibility. Additionally, the diagnostic reagents produced consistent reaction outcomes across the PCR instrument, metal bath, and water bath (**Figure 7**).

### 3.7 Detection of clinical samples by the dual LAMP-LFD method

A total of 106 clinical samples, including pleural effusion, lung tissue, nasal swabs, and others collected from pigs, were tested using the established dual LAMP-LFD method, as well as standard reference methods. The results showed a positive rate of 11.32% (12/106) for *S. suis*, 25.47% (27/106) for *G. parasuis*, and a mixed infection rate of 2.83% (3/106). Different levels of positivity were detected for *S. suis* and *G. parasuis* in various tissues, including serum, heart, liver, spleen, lungs, kidneys, tonsils, thymus, and abdominal and femoral lymph nodes. The results were consistent with those obtained using national standard detection methods, confirming the accuracy of the dual LAMP-LFD method (**Table 3**).

**Table 3:** Detection positive rate of clinical samples and comparison with reference methods.

Pathogens	Dual LAMP-LFD assay	Reference method*	Agreement
	Positive	Positive	
*S. suis*	11.32% (12/106)	11.32% (12/106)	100%
*G. parasuis*	25.47% (27/106)	25.47% (27/106)	100%
*S. suis* + *G. parasuis*	2.83% (3/106)	2.83% (3/106)	100%

*GB/T 19915.5—2005, Protocol of multiplex PCR identification of *Streptococcus suis* type 2; GB/T 34750—2017, Detection methods for *haemophilus parasuis.*

## 4 Discussion

Streptococcal and Glässer’s diseases pose significant challenges to the livestock industry, with annual increases in the incidence of bronchopneumonia caused by *S. suis* and *G. parasuis* of approximately 6% and 4.3%, respectively, along with a 23% annual rise in *S. suis*-related endocarditis (Silva et al., 2023). Surveys conducted in China between 2017 and 2021 reported detection rates of 63.50% for *S. suis* and 28.54% for *G. parasuis* (Sun et al., 2022). *S. suis* and *G. parasuis* was found to be major pathogens of porcine respiratory disease in Guangxi province, with prevalence rates of 65.21% and 48.19%, respectively, and a mixed infection rate of 13.10% (Rao et al., 2023). These statistics highlight the urgent need for rapid and effective diagnostic methods to manage and control these infections. The dual LAMP-LFD method developed in this study addresses this need by offering advantages such as simplicity, rapid results, ease of use, and no requirement for specialized equipment or personnel. These features make it particularly suitable for on-site diagnosis of *S. suis* and *G. parasuis* infections, enabling timely decisions regarding treatment and vaccination.

The selection of target genes is a key factor in effective LAMP detection. In this study, the *S. suis gdh* gene and the *G. parasuis infB* gene were chosen for the dual LAMP-LFD method. The *gdh* gene encodes glutamate dehydrogenase, a key virulence factor, and exhibits high nucleotide sequence conservation among different *S. suis* serotypes (96% to 100%) (Okwumabua et al., 2001; Okwumabua et al., 2003; Xu et al., 2021). While 16S rRNA is commonly used for *G. parasuis* detection, it lacks specificity in distinguishing *G. parasuis* from closely related species such as *Actinobacillus (* Turni et al., 2010; Yang et al., 2010; Zhang et al., 2012). In contrast, the *infB* gene, as reported by Hedegaard et al., serves as a reliable genetic marker for species identification and can effectively distinguish *G. parasuis* from related species (Hedegaard et al., 2000). Turni et al. and Pilchová et al. confirmed the suitability of the *infB* gene for real-time fluorescent quantitative PCR and LAMP detection methods, enhancing the specificity of the dual LAMP-LFD method (Turni et al., 2010; Pilchova et al., 2020).

The design and optimization of LAMP primers were critical to the success of this method. Initial primer selection was performed using Primer Explorer V5 software, followed by screening with a quantitative PCR instrument to identify primers that exhibited optimal performance, as indicated by lower Ct values, smoother curves, earlier peaks, and the absence of non-specific amplification. Optimization revealed that a reaction temperature of 62°C and a primer concentration ratio of 1:4 provided the best amplification results. Additionally, a 40 minute reaction time effectively stabilized amplification products.

Previous studies have reported varying detection limits for LAMP methods. Li et al. achieved a detection limit of 1 fg for the *S. suis ermB* and *mefA* genes (Li et al., 2022). Zhang et al. found that gel electrophoresis and SYBR Green I methods for *S. suis* type 2 *cps2J*-LAMP products demonstrated the highest sensitivity, with a detection limit of 7.16 copies/μL, although dye methods are prone to non-specific amplification (Zhang et al., 2013; Guang et al., 2023). Pilchová et al. established a LAMP method for *G. parasuis infB* with a detection limit of 10 fg/μL, while Guang et al. developed a LAMP-LFD method for the same gene with a detection limit of 1.285 × 10^-^¹² ng/μL (Silva et al., 2023). Unlike these methods, which are limited to single-pathogen detection, the dual LAMP-LFD method developed here can simultaneously detect both *S. suis* and *G. parasuis*, with minimum detection limits of 22 and 18 copies/μL for recombinant plasmids, respectively. The consistency of results obtained from testing 106 clinical samples compared to national standard methods confirms the high sensitivity and effectiveness of the method for clinical application. *S. suis* and *G. parasuis* were detected in tissues such as the lungs, tonsils, and blood of pigs, underscoring the importance of enhanced health monitoring in pig herds. Particular attention should be given to *S. suis* due to its potential zoonotic risk, especially in high-exposure environments such as slaughterhouses.

## 5 Conclusion

A rapid and convenient dual LAMP-LFD detection method for the simultaneous identification of *S. suis* and *G. parasuis* has been successfully developed and validated. This method exhibits high sensitivity, strong specificity, and excellent reproducibility. Preliminary testing of clinical samples yielded promising results, confirming the method’s effectiveness. This study presents a novel diagnostic tool that enhances the rapid identification and epidemiological surveillance of *S. suis* and *G. parasuis*, offering significant advantages for the prevention, control, and timely treatment of streptococcal and Glässer’s diseases.

## Data Availability

The original contributions presented in the study are included in the article/**Supplementary Material**. Further inquiries can be directed to the corresponding authors.
