# Supplementary material for: Development and application of a dual LAMP-LFD assay for the simultaneous detection of Streptococcus suis and Glaesserella parasuis
Source: Front Cell Infect Microbiol. 2025 Apr 1;15:1575365. doi: 10.3389/fcimb.2025.1575365 (PMC11996922; doi:10.3389/fcimb.2025.1575365)
Supplement: Supplementary file 1 [file DataSheet1.pdf]

Supplementary materials

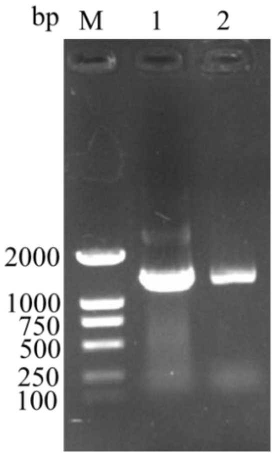

Figure S1 PCR amplification of recombinant plasmids pMD-SS and pMD-GPS

M: DL 2000DNA Marker；1: pMD-SS；2: pMD-GPS；

Table S1 Liquid counts of *S. suis* and *G. parasuis*.

	No.	10^{-5}	10^{-6}	10^{-7}
S. suis	1	203	12	2
	2	185	23	1
	3	166	14	2
	mean	185	16	2
G. parasuis	1	789	84	6
	2	374	36	2
	3	622	60	6
	mean	595	60	5
